# Psychological ownership and academic identity: unveiling their roles in promoting teaching innovation

**DOI:** 10.3389/fpsyg.2026.1833248

**Published:** 2026-05-15

**Authors:** Luote Dai, Saizhi Wang, Jiajia Chen

**Affiliations:** 1 School of Digital Economy & Trade, Wenzhou Polytechnic, Wenzhou, China; 2 School of General Education, Wenzhou Polytechnic, Wenzhou, China

**Keywords:** academic identity, integration of research and teaching, perceived organizational support, psychological ownership of research, teaching innovation

## Abstract

**Introduction:**

Teaching innovation is increasingly important in higher education, yet the psychological mechanisms through which faculty members integrate research and teaching remain insufficiently understood. This study investigates the relationship between psychological ownership of research and teaching innovation, focusing on the mediating role of academic identity and the moderating effects of perceived organizational support and professional identity.

**Methods:**

Data were collected from 452 faculty members across research universities, applied universities, and vocational colleges in China. Structural equation modeling was used to test the hypothesized mediation and moderation effects, as well as institutional differences among university types.

**Results:**

The results show that psychological ownership of research does not directly affect teaching innovation. However, it significantly promotes teaching innovation indirectly through academic identity. Perceived organizational support strengthens the relationship between psychological ownership of research and academic identity. In contrast, professional identity does not significantly moderate the relationship between academic identity and teaching innovation. Further analysis reveals institutional differences, with stronger effects observed in research-oriented universities.

**Discussion:**

These findings highlight the importance of academic identity as a psychological pathway linking research engagement to teaching innovation. They also suggest that organizational support can enhance faculty members' identification with their academic roles, thereby promoting innovative teaching practices. The study contributes to understanding how psychological and institutional factors shape teaching innovation and offers practical implications for higher education institutions seeking to support the integration of research and teaching.

## 1 Introduction

In the context of the global knowledge economy and the transformation of higher education, the deep integration of research and teaching has become a key issue in promoting the improvement of educational quality and the professional development of teachers (Darling-Hammond, 2021). With the popularization of the concept of “research-based teaching”, teachers are no longer merely transmitters of knowledge, but are expected to form a dynamic interaction between research and teaching, enabling students to be exposed to cutting-edge knowledge during the learning process and develop critical thinking and innovation abilities (Healey et al., 2016). This “integration of science and education” not only helps to enhance the academic and practical nature of the curriculum content, but also provides a new source of impetus for the development of teachers themselves (Gibbs et al., 2022). However, despite the continuous emphasis on the integration of research and teaching at both the policy and practical levels, the psychological motivations and identity construction mechanisms demonstrated by teachers in this process have not been fully revealed.

Existing research generally emphasizes that scientific research can directly promote Teaching Innovation. The knowledge and methodological experience that teachers acquire through research often enter the classroom through curriculum updates, case studies, and inquiry-based learning, thereby enhancing teaching quality (Constantinou et al., 2018). At the same time, research experience can also strengthen teachers' sense of authority and self-efficacy in the classroom, making them more willing to try new teaching strategies (Morris and Usher, 2011). This “from research to classroom” approach provides an important empirical basis for understanding the integration of science and education, but an overemphasis on knowledge transfer often neglects the psychological and identity role that teachers play in this process.

Academic Identity has become an important perspective for understanding the research teaching nexus because it reflects whether teachers can integrate their researcher and educator roles into a coherent academic self (Billot, 2010). Teachers who internalize research as part of this academic self are more likely to carry research based values and knowledge into teaching, whereas weak identity integration may reduce teaching engagement and innovation (Huang et al., 2019).

Psychological ownership offers a useful lens for explaining this process because it captures how feelings of responsibility, control, and self investment toward one's own research may shape teachers' academic self understanding (Dawkins et al., 2017). In higher education, Psychological Ownership of Research can strengthen teachers' responsibility for and control over their own research work, thereby supporting the formation of a more stable Academic Identity (Xue et al., 2024). When translated into Academic Identity, such ownership may further connect research engagement with Teaching Innovation (Cocieru et al., 2021). However, this identity based transformation process remains insufficiently clarified.

Although the research teaching nexus has been widely discussed in higher education, existing studies still tend to explain teaching innovation through a relatively direct logic of knowledge transfer, assuming that research experience naturally flows into the classroom in the form of updated content, stronger methodological competence, or greater instructional confidence (Karkkainen et al., 2023). This perspective is important, but it leaves a key question insufficiently answered: why do some teachers with substantial research engagement actively translate research into innovative teaching, whereas others do not, even under broadly similar institutional conditions? The unresolved issue is therefore not simply whether research matters for teaching, but through what psychological mechanism research related investment is converted into classroom innovation. In particular, prior work has paid growing attention to identity in the research teaching nexus, yet it has not clearly specified why academic identity, rather than a broader professional identity, should serve as the pivotal transformation mechanism. Academic identity refers to teachers' integrated understanding of themselves as both researchers and educators, whereas professional identity more broadly reflects commitment to the teaching profession and its values. Conflating the two risks obscuring the distinct process through which research related psychological states are translated into teaching practice.

To address this gap, the present study examines how psychological ownership of research influences teaching innovation through the formation of academic identity, while also considering the boundary roles of perceived organizational support and professional identity. By doing so, this study aims to make three contributions. First, it extends research on the research teaching nexus by challenging the implicit linear assumption that research related motivation or investment automatically produces teaching innovation. Instead, it argues that psychological ownership of research must first be transformed into an integrated academic self before it can shape innovative teaching behavior. Second, it clarifies the theoretical boundary between academic identity and professional identity by positioning the former as a role integration mechanism that links research and teaching, and the latter as a broader value based orientation whose function may be more limited in activating specific innovative practices. Third, it situates this identity based process within organizational context, showing that whether research related psychological ownership becomes pedagogically meaningful depends not only on individual motivation, but also on the institutional conditions that support identity construction. In this way, the study does more than confirm expected associations. It refines how identity theory and psychological ownership theory can jointly explain when and how research becomes teaching innovation in higher education.

## 2 Literature review and hypothesis analysis

The interaction of teachers between research and teaching has long been one of the focuses of higher education research. Traditional research has mostly been conducted from the perspective of “research promoting teaching”, arguing that research can directly drive the renewal of curriculum content and the improvement of teaching methods (Volkova, 2018; Serdyukov, 2017). However, with the increasing attention to the individual psychology and identity construction of teachers, the academic community has gradually realized that the benign interaction between scientific research and teaching not only relies on the explicit knowledge transfer, but is also deeply rooted in the psychological ownership and Academic Identity construction process of teachers (Dawkins et al., 2017). Therefore, this paper proposes a chain mechanism framework of “Psychological Ownership of Research—Academic Identity—Teaching Innovation”, and develops research hypotheses on this basis.

### 2.1 Psychological ownership of research and teaching innovation

In the context of higher education, whether teachers engage in Teaching Innovation depends not only on external institutional arrangements and resource provision, but also on their internal psychological drivers. In this study, Psychological Ownership of Research refers to teachers' subjective sense that the research agenda they develop, the projects they pursue, the research process they shape, and the outputs they produce are personally “theirs.” It reflects a feeling of responsibility, control, and self-investment toward one's own research work rather than ownership of an academic discipline in a broad or abstract sense (Pierce et al., 2001). When teachers perceive their research as a meaningful extension of the self, they are more likely to treat research not merely as an external job requirement, but as a personally significant domain of academic work.

This sense of ownership is often associated with stronger motivational engagement. Self determination theory suggests that when individuals perceive a task as closely connected to the self, they experience greater autonomy and competence, which in turn stimulates sustained effort and improvement oriented behavior (Ryan and Deci, 2000; Gagne et al., 2022). In the present context, when teachers see research topics, processes, and outputs as extensions of their self worth, they may become more motivated to bring research based thinking into the classroom rather than leaving it confined to the research domain. Such motivation can strengthen the coupling between research and teaching and provide continuous psychological energy for Teaching Innovation (Avey et al., 2009).

Beyond motivation, Psychological Ownership of Research may also foster the accumulation of resources that are transferable to teaching. Teachers who are deeply invested in their research are more likely to develop problem awareness, methodological sensitivity, and evidence based reasoning through the research process (Kyza and Georgiou, 2025). These capabilities can be translated into teaching practice through curriculum renewal, interdisciplinary case design, research based learning activities, and other innovative pedagogical forms (Brew, 2010). In this sense, psychological ownership not only increases teachers' willingness to innovate, but may also provide the cognitive and skill based foundation needed to support innovation in teaching.

In addition, psychological ownership has been shown to stimulate proactive behavior, making individuals more inclined to reshape their work and environment in order to optimize outcomes (Chen et al., 2023). For teachers, this proactive tendency may be reflected in curriculum reconstruction, resource integration, and the adoption of research oriented teaching methods (Webb, 2009). When teachers feel strong ownership of research, they may be more willing to externalize academic values in classroom practice and encourage deeper student learning and inquiry (Bhardwaj et al., 2025). Although the influence of research related ownership on Teaching Innovation may not always be direct, it is still theoretically reasonable to expect a positive association between the two. Therefore, the following hypothesis is proposed:

H1: Psychological Ownership of Research positively influences Teaching Innovation.

### 2.2 Psychological ownership of research and academic identity

Although psychological ownership initially describes a felt relationship between the individual and a valued object, its implications often extend beyond task engagement to identity formation. Psychological ownership reflects a subjective sense that a target is “mine,” and this perception is typically accompanied by heightened responsibility, control, and self investment (Pierce et al., 2001; Dawkins et al., 2017). In the context of higher education, when teachers develop strong psychological ownership of their own research work, they are not merely participating in research activities as routine job requirements. Rather, they are more likely to perceive their self developed research agenda, ongoing projects, research processes, and scholarly outputs as personally meaningful extensions of their academic selves, rather than viewing ownership in terms of an academic discipline as a whole. This meaning laden attachment can gradually shape how they understand their place within the academic community.

This process is especially important because university teachers are not defined by a single professional role. They are expected to function simultaneously as researchers and educators, and the way they integrate these two roles is central to their sense of self in higher education (Henkel, 2005; Billot, 2010). In this study, Academic Identity refers to teachers' self understanding as academic actors who integrate research and teaching into a coherent role orientation. This construct is narrower and more role specific than general professional identity. Whereas professional identity reflects commitment to teaching as a profession and to broader educational values (Canrinus et al., 2012), academic identity focuses more directly on whether teachers perceive themselves as legitimate members of an academic community in which research and teaching are jointly meaningful (Henkel, 2005; Billot, 2010). Therefore, if psychological ownership of research strengthens teachers' attachment to research as part of the self, it should also increase the likelihood that they internalize a stronger academic identity.

This positive relationship can be understood through at least three mechanisms. First, psychological ownership encourages internalization. When teachers experience research as personally theirs, they are more likely to move from performing research to identifying with research as part of who they are (Pierce et al., 2001; Brown and Everson, 2019). Second, ownership can stimulate deeper participation in academic interaction. Teachers who feel responsible for their research are often more willing to seek feedback, exchange ideas, and engage with peers, and these interactions help reinforce their recognition as academic contributors (Khasawneh et al., 2023). Third, psychological ownership provides narrative continuity. Because teachers attach personal meaning to their research activities, they are better able to construct a stable self narrative in which research and teaching are not fragmented demands but mutually reinforcing aspects of academic life (Huang et al., 2025).

Taken together, psychological ownership of research should not remain confined to momentary motivation or task level engagement. It is likely to shape how teachers define themselves as academic actors, thereby strengthening academic identity. Thus, the following hypothesis is proposed:

H2: Psychological Ownership of Research positively influences Academic Identity.

### 2.3 Academic identity and teaching innovation

Teaching Innovation is generally understood as the creative and improvement oriented behaviors demonstrated by teachers in course objective setting, content organization, pedagogical methods, technology integration, and classroom interaction (Ketelaar et al., 2012; Shermukhammadov, 2022; Tripon, 2026). In practice, this may be manifested as updating course content with recent research, redesigning curriculum structures, adopting inquiry based, project driven, or flipped learning approaches, integrating digital tools into instruction, and strengthening the authenticity of learning through interdisciplinary cases and practical tasks (Cloonan et al., 2019; Tripon, 2026).

Academic identity is highly relevant here because it reflects whether teachers see research and teaching as integrated rather than competing domains of work. Prior research suggests that the research teaching nexus becomes more productive when teachers do not treat research as detached from teaching, but instead regard both as parts of a unified academic mission (Henkel, 2005; Billot, 2010; Kaasila et al., 2021). Teachers with a stronger academic identity are therefore more likely to perceive the classroom as an appropriate site for enacting academic values, extending research based reasoning, and bringing intellectual inquiry into pedagogy. In this sense, academic identity provides a role based lens through which teaching is interpreted not as routine delivery, but as a meaningful arena for academic expression and innovation.

The effect of academic identity on teaching innovation can be explained through cognitive, motivational, and social mechanisms. Cognitively, teachers with stronger academic identity are more likely to frame teaching as knowledge creation and knowledge translation rather than simple content transmission, which encourages them to update materials and adopt inquiry oriented approaches (Henkel, 2005; Stanovich, 2003). Motivationally, academic identity can generate a stronger sense of mission and pride, making teachers more willing to invest in experimentation and pedagogical change (Yang et al., 2022). Socially, academic identity is often reinforced through academic communities, and teachers who feel recognized as academic members may be more inclined to embody those values in classroom practice, thereby promoting innovative teaching (Mehdizadeh et al., 2024; Strom and Martin, 2022).

Importantly, academic identity is conceptually different from professional identity in this pathway. Professional identity reflects a broader commitment to teaching as a valued profession, whereas academic identity more directly captures whether teachers integrate the roles of researcher and educator. Because teaching innovation in this study is examined in the context of the research teaching nexus, the academic role integration embedded in academic identity should be especially relevant. When teachers view themselves as scholars who teach and teachers who research, they are more likely to introduce current knowledge, research methods, and exploratory thinking into instructional practice.

Accordingly, the following hypothesis is proposed:

H3: Academic Identity positively influences Teaching Innovation.

### 2.4 The mediating role of academic identity

The preceding arguments suggest that psychological ownership of research may stimulate teaching innovation and that academic identity is positively associated with innovative teaching. However, the relationship between research related ownership and teaching innovation is unlikely to be purely direct. A teacher may feel highly responsible for and emotionally attached to research, yet still keep that investment within the research domain unless it becomes incorporated into a broader understanding of the self as an academic who integrates research and teaching. This is why academic identity is theorized here not merely as an associated factor, but as the key transformation mechanism linking psychological ownership of research to teaching innovation.

This mediating logic is consistent with both psychological ownership theory and identity based perspectives. Psychological ownership generates attachment, responsibility, and self relevance toward an object or activity (Pierce et al., 2001; Dawkins et al., 2017), but such feelings do not automatically dictate cross domain behavior. For research related ownership to shape classroom practice, teachers must first interpret research as part of their enduring academic role. Identity theory suggests that behavior is more likely to follow from self meanings when those meanings become integrated into role based self definitions (Henkel, 2005; Hughes, 2010). In the present context, this means that psychological ownership of research should promote teaching innovation chiefly when it strengthens academic identity, which then guides teachers to express research based values and practices in the classroom.

This argument also helps clarify why academic identity, rather than professional identity, is the central mediator in the model. Professional identity reflects broad commitment to education and the teaching profession (Canrinus et al., 2012), but it does not necessarily capture whether research related experiences are incorporated into the self. Academic identity is more directly relevant because it concerns the integration of researcher and educator roles. Since the present study focuses on how research based psychological experience becomes pedagogically meaningful, academic identity is conceptually the more proximal mechanism.

Existing scholarship on the research teaching nexus and teacher identity provides indirect support for this mediation logic. When teachers experience stronger alignment between their research activities and academic role understanding, they are more likely to bring research into teaching practice and to innovate in ways that reflect scholarly values (Billot, 2010; Kaasila et al., 2021; McCune, 2021). By contrast, if research remains psychologically important but identity integration is weak, teachers may continue to invest in research without translating that investment into teaching innovation.

Therefore, academic identity is expected to function as the explanatory bridge through which psychological ownership of research influences teaching innovation. Thus, the following hypothesis is proposed:

H4: Academic Identity mediates the relationship between Psychological Ownership of Research and Teaching Innovation.

### 2.5 The moderating roles of perceived organizational support and professional identity

Although the transformation from psychological ownership of research to teaching innovation is expected to occur through academic identity, this process is unlikely to unfold independently of context. Teachers are embedded in organizational environments that can either facilitate or constrain the conversion of psychological experience into identity and behavior. For this reason, the present study further considers the moderating roles of Perceived Organizational Support and Professional Identity.

First, perceived organizational support should strengthen the relationship between psychological ownership of research and academic identity. Perceived organizational support refers to the extent to which teachers believe that their institution values their contributions and cares about their development (Eisenberger et al., 1986, 2020). When such support is high, teachers are more likely to feel that their research efforts are recognized and institutionally legitimized. In that case, ownership of research is not experienced as a purely private attachment, but as something that can be meaningfully incorporated into one's academic role within the organization. Institutional encouragement, resource provision, and cultural recognition may therefore amplify the degree to which research ownership is translated into academic identity (Karkkainen et al., 2023; Hassan et al., 2024; Kilinc et al., 2024). By contrast, when perceived organizational support is low, even teachers who feel strong ownership of their research may find it harder to develop a stable academic identity because the surrounding environment does not reinforce that self understanding.

Accordingly, the following hypothesis is proposed:

H5: Perceived Organizational Support moderates the relationship between Psychological Ownership of Research and Academic Identity, such that the positive relationship is stronger when Perceived Organizational Support is higher.

Second, professional identity is expected to moderate the relationship between academic identity and teaching innovation. Professional identity reflects teachers' broader commitment to the teaching profession, including their endorsement of educational values, sense of mission, and pride in being a teacher (Canrinus et al., 2012). Although academic identity is the more proximal mechanism linking research and teaching, the extent to which this identity is enacted in innovative teaching may still depend on whether teachers place strong value on teaching as a profession. When professional identity is high, teachers who already see themselves as integrated academic actors may be more willing to translate that role understanding into concrete instructional change, because they attach greater importance to improving teaching practice and fulfilling educational responsibilities. In contrast, when professional identity is weaker, academic identity may remain oriented more toward scholarly self understanding than toward pedagogical enactment.

Based on this reasoning, the following hypothesis is proposed:

H6: Professional Identity moderates the relationship between Academic Identity and Teaching Innovation, such that the positive relationship is stronger when Professional Identity is higher.

**Figure 1:**
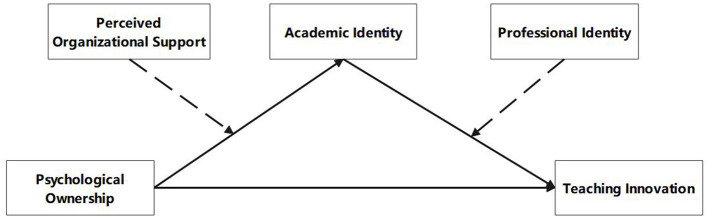
Research framework.

The framework of the study is shown in Figure 1:

## 3 Methods

### 3.1 Research design

In order to test the theoretical model and research hypothesis proposed in the study, this paper adopts a research method combining questionnaire survey and empirical modeling. The overall design was a cross-sectional survey, and the data were analyzed through the structural equation model. The cross-sectional design can collect large sample data at the same point in time, more efficiently reflecting the interaction mechanism between teachers' research and teaching, and is also suitable for systematic testing of mediating and moderating effects.

The subjects were selected from a group of teachers in universities in eastern, central and western China, covering both undergraduate and higher vocational education stages, to ensure the diversity and representativeness of the sample. To minimize the interference caused by regional and institutional differences, the study used stratified sampling, dividing by geographical region and institution type, and randomly selecting teachers from each layer as samples. Based on the basic requirements for sample size of the structural equation model (Kline, 2023), this study plans to collect approximately 500 questionnaires and expects about 450 valid samples, which can meet the needs of parameter estimation and hypothesis testing. All participants are required to have dual experience in research and teaching and to participate voluntarily.

Variables were measured using internationally mature scales and revised in the context of higher education. Psychological Ownership of Research was adapted from the scales of Pierce et al. (2001) and Avey et al. (2009) to capture teachers' felt ownership of their own research work, including responsibility for research outcomes, control over the research process, and the personal significance of research success or failure. Academic Identity was adapted from the work of Henkel (2005) and Billot (2010), with items designed to capture the integration of researcher and educator identities, such as “I can effectively integrate research results into teaching”; Teaching Innovation is adapted from the scale of Ketelaar et al. (2012), with items such as “I often try new teaching methods in the classroom.” Teaching Innovation was measured as teachers' self reported innovative efforts in curriculum renewal, pedagogical methods, technology use, and classroom practice. The items covered behaviors such as trying new teaching methods, introducing recent research findings into teaching, designing interdisciplinary cases, and using information technology to improve instruction. Perceived Organizational Support is adapted from the Perceived Organizational Support Scale of Eisenberger et al. (1986) and Eisenberger et al. (2020) to reflect the degree of support that teachers receive in scientific research and teaching. The Professional Identity scale draws on the relevant research of Canrinus et al. (2012) to design items that reflect educational mission and professional value commitment. All items were on a 7-point Likert scale, ranging from “strongly disagree” to “strongly agree”. In addition, demographic variables such as gender, age, job title, years of teaching, and type of institution were controlled.

Before the formal investigation, the study tested the comprehensibility and reliability of the questionnaire items through a small-scale pretest (*n* = 30), and invited five scholars with research experience to evaluate the content validity. The revised formal questionnaire was distributed through an online platform and teacher communities, supplemented by paper questionnaires at some institutions. To ensure data quality, the questionnaire includes attention test questions and limits the time range for filling in. All participants signed the informed consent form before filling it out, and the study was approved by the school ethics committee (approval number: WZPTEC-2025-026).

Data analysis is divided into three stages. The first stage involves reliability and validity tests, calculating Cronbach's α coefficient to test internal consistency, and using confirmatory factor analysis to test aggregated validity and discriminant validity. In the second stage, a structural equation model is constructed to evaluate the overall fit of the model (χ^2^/df, CFI, TLI, RMSEA, etc.) and to examine the path relationship among Psychological Ownership of Research, Academic Identity and Teaching Innovation. The third stage focuses on the mediating and moderating effects: the Bootstrap method is used to test the mediating role of Academic Identity, and the moderating effects of Perceived Organizational Support and Professional Identity are examined through interaction term regression and multi-group analysis. All data analysis will be conducted in SPSS 26.0 and AMOS 24.0 software to ensure the robustness of the results.

### 3.2 Data collection

In this study, a self-screening question was included at the beginning of the questionnaire to confirm respondents' eligibility: “Have you engaged in both teaching and research activities in the past 3 years? If not, please refrain from completing this questionnaire.” This ensured that all participants had relevant experience with both teaching and research, which is essential for the present study. Data collection was conducted between March and May 2025. A total of 486 responses were collected, of which 452 were valid after removing incomplete and inattentive responses.

Table 1 summarizes the demographic characteristics of the final sample. Among the respondents, 54.6% were male (*n* = 247) and 45.4% were female (*n* = 205). In terms of age, the largest group was 30–39 years (42.7%) followed by 20–29 years (28.8%). The majority of participants held either a Master 's degree (51.1%) or a Doctoral degree (23.3%) while 25.6% had a Bachelor's degree. Regarding academic rank, 28.5% were assistant lecturers, 36.1% were lecturers, 23.7% were associate professors and 11.7% were full professors. Participants were distributed across different types of institutions including research universities (41.4%), teaching-oriented universities (32.3%), and vocational colleges (26.3%). Average teaching experience was 9.3 years with nearly half of the respondents reporting that they regularly combined teaching with research.

**Table 1:** Demographic characteristics of the response sample (*n* = 452).

Characteristics	Frequency	Percentage (%)
Gender		
Male	247	54.6
Female	205	45.4
Age		
29 years old and under	130	28.8
30–39	193	42.7
40–49 years old	93	20.6
50 years old and above	36	7.9
Education		
Bachelor	116	25.6
Master	231	51.1
Doctor	105	23.3
Title		
Assistant lecturer	129	28.5
Lecturer	163	36.1
Associate professor	107	23.7
Professor	53	11.7
Institution type		
Research universities	187	41.4
Teaching university	146	32.3
Higher vocational colleges	119	26.3

### 3.3 Common method variance test

Since all variables in this study were measured by self-reporting questionnaires and obtained from the same source, there is a possibility that Common Method Variance (CMV) will interfere with the results of the study. Therefore, the CMV needs to be tested before entering the structural modeling to rule out systematic errors due to consistency in measurement methods. First, Harman's single-factor test was conducted. All measurement items were simultaneously included in the non-rotating exploratory factor analysis (EFA), and the results showed that the first factor accounted for only 28.764% of the total variance, far below the 50% critical threshold, initially ruling out the possibility of single-factor effect dominance. Secondly, to more accurately identify potential CMV issues, this study further validated using the Unmeasured Latent Method Construct (ULMC) model. A latent method factor was introduced into the original confirmatory factor analysis (CFA) model, and all measurement items were simultaneously loaded onto the method factor while retaining their relationship with the original latent variables. The fitting results show: The original CFA model fits well (χ^2^/df = 2.417, CFI = 0.946, RMSEA = 0.051, SRMR = 0.046), and the method factor model fits slightly better (χ^2^/df = 2.301, CFI = 0.952, RMSEA = 0.048, SRMR = 0.044). However, the difference in χ^2^ between the two was 14.832 (df difference = 11), and the change in CFI was only 0.006, not exceeding the substantial improvement threshold of 0.010. The results from both methods suggest that common method variance does not appear to be a major concern in this study, although it cannot be fully ruled out.

### 3.4 Model measurement

In the validation of the measurement model, factor loadings for each observation item were first tested. As shown in Table 2, the standardized factor loadings for all items were between 0.794 and 0.913, significantly above the threshold of 0.700, indicating that each measurement item could well reflect its corresponding latent variable (Hair et al., 2016).

**Table 2:** Factor loadings and reliability/validity statistics for each construct.

Constructs	Item label	Standardized loading	CR	Cronbach's α	AVE
Psychological ownership of research (PO)	PO1: I feel that research work is an integral part of my academic career	0.842	0.910	0.882	0.662
	PO2: I have a strong sense of responsibility for research results	0.873			
	PO3: I regard scientific research as an important part of my personal Academic Identity	0.857			
	PO4: I have a strong sense of control over the research process	0.811			
	PO5: I think the success or failure of research is closely related to me personally	0.826			
Academic identity (AI)	AI1: I am both a researcher and an educator in the academic community	0.794	0.923	0.901	0.685
	AI2: I identify myself as a scholar rather than just a teacher	0.827			
	AI3: I am capable of effectively integrating research results into teaching	0.864			
	AI4: I integrate my researcher identity with my educator identity	0.813			
	AI5: I often embody the values of the academic community in my teaching	0.842			
	AI6: I have felt the shaping of my Academic Identity on my teaching methods	0.851			
Teaching innovation (TI)	TI1: I often try out new teaching methods in the classroom	0.885	0.936	0.914	0.708
	TI2: I will introduce the latest research findings into my classes	0.902			
	TI3: I encourage students to acquire knowledge through inquiry-based learning	0.876			
	TI4: I will design interdisciplinary teaching cases	0.849			
	TI5: I am willing to use information technology to improve teaching	0.913			
	TI6: I try to constantly adjust the curriculum based on student feedback	0.895			
Perceived organizational support (OS)	OS1: My school values my contributions to research and teaching	0.816	0.884	0.857	0.653
	OS2: My school has provided me with sufficient resources for my research and teaching	0.844			
	OS3: My school cares about my well-being at work	0.833			
	OS4: My school encourages me to be innovative in teaching	0.809			
Professional identity (PI)	PI1: Teaching is an indispensable part of my life	0.871	0.902	0.874	0.692
	PI2: I firmly believe that the teaching profession is of great significance to social development	0.896			
	PI3: I'm proud to be a teacher	0.874			
	PI4: I consider teaching as an important part of my identity	0.832			
	PI5: I have a long-term commitment to the education profession	0.854			

Further examination of reliability and convergent validity revealed that the compound reliability (CR) of Psychological Ownership of Research (PO), Academic Identity (AI), Teaching Innovation (TI), Perceived Organizational Support (OS), and Professional Identity (PI) were 0.910, 0.923, 0.936, 0.884, and 0.902 respectively, all greater than 0.700. Cronbach's α values were 0.882, 0.901, 0.914, 0.857, and 0.874 respectively, all above 0.800; The average variance extracted (AVE) was between 0.653 and 0.708, all above the standard of 0.500. The results suggest that the constructs have good internal consistency and convergent validity.

To further validate discriminant validity, the Fornell-Larcker criterion was used in this study. The results are shown in Table 3, where the AVE square roots of all constructs are greater than their correlation coefficients with other constructs. For instance, the AVE square root of Psychological Ownership of Research is 0.814, which is higher than its correlation coefficients with Academic Identity (0.576), Teaching Innovation (0.552), Perceived Organizational Support (0.488), and Professional Identity (0.471). The AVE square root of Academic Identity is 0.828, which is also greater than its correlation coefficient with other constructs. Therefore, the constructs demonstrate satisfactory discriminant validity at the statistical level. At the same time, Academic Identity and Professional Identity are theoretically adjacent constructs and therefore require clear conceptual differentiation. In this study, Academic Identity refers to the integration of researcher and educator roles, whereas Professional Identity reflects broader commitment to teaching as a profession.

**Table 3:** Square roots of AVE and correlations among latent variables.

Constructs	PO	AI	TI	OS	PI
PO	0.814				
AI	0.576	0.828			
TI	0.552	0.609	0.842		
OS	0.488	0.517	0.539	0.808	
PI	0.471	0.553	0.587	0.526	0.832
VIF	2.371	2.654	3.082	1.963	2.417

Meanwhile, to eliminate multicollinearity interference, the variance inflation factor (VIF) of the latent variable was calculated. The results showed that the VIF values of the five constructs ranged from 1.963 to 3.082 (with an average of 2.497), well below the empirical threshold of 10 and also below the strict standard of 5, indicating that there was no serious multicollinearity problem in the study data.

## 4 Results

### 4.1 Path analysis

The path analysis results of the structural model are shown in Table 4. The direct effect of PO on TI did not reach a significant level [H1: β = 0.094, *t* = 1.271, *p* = 0.204, 95% CI (−0.054, 0.236)], indicating that PO cannot directly predict TI. In contrast, PO has a significant positive impact on AI [H2: β = 0.482, *t* = 8.213, *p* < 0.001, 95% CI (0.365, 0.574)], while the effect of AI on TI is equally significant [H3: β = 0.514, *t* = 9.026, *p* < 0.001, 95% CI (0.403, 0.605)]. Further mediating effect tests showed that PO had a significant indirect effect on TI through AI [H4: β = 0.248, *p* < 0.001, 95% CI (0.176, 0.335)], with no zero confidence interval, indicating that AI played a full mediating role between PO and TI.

**Table 4:** Path analysis results.

Assumptions	Path relationship	Beta.	*t*-value	*p*-value	95% CI	Conclusion
H1	PO → TI (direct)	0.094	1.271	0.204	(−0.054, 0.236)	Not supported
H2	PO → AI	0.482	8.213	< 0.001	(0.365, 0.574)	Support
H3	AI → TI	0.514	9.026	< 0.001	(0.403, 0.605)	Support
H4	PO → AI → TI (mediating effect)	0.248	-	< 0.001	(0.176, 0.335)	Support
H5	OS × PO → AI	0.143	2.684	0.007	(0.047, 0.232)	Support
H6	PI × AI → TI	0.082	1.744	0.081	(−0.011, 0.181)	Not supported

In terms of moderating effects, OS plays a significant positive moderating role in the “PO → AI” pathway [H5: β = 0.143, *t* = 2.684, *p* = 0.007, 95% CI (0.047, 0.232)], suggesting that PO has a stronger effect on AI in high organizational support scenarios. However, the moderating effect of PI on “AI → TI” did not reach a significant level [H6: β = 0.082, *t* = 1.744, *p* = 0.081, 95% CI (−0.011, 0.181)], suggesting that the moderating effect of PI was not significant in the sample of this study.

Taken together, the path analysis suggests that the effect of Psychological Ownership of Research on Teaching Innovation is primarily transmitted through Academic Identity. Perceived Organizational Support strengthens this process, whereas the moderating role of Professional Identity was not supported.

### 4.2 Moderating mediation analysis with total effect breakdown

After examining the direct effect and mediating effect, this study further investigated the role of OS and PI in the mediating path, and decomposed the total effect from PO to TI.

The conditional indirect effects of the mediating path of “PO → AI → TI” at different moderating variable levels were estimated using 5,000 bias-corrected bootstraps. As shown in Table 5, Perceived Organizational Support has a significant moderating effect on indirect effects. When OS was at a low level (−1 SD), the conditional indirect effect was β = 0.174, 95% CI (0.102, 0.256); When OS was at the average level, the conditional indirect effect was β = 0.248, 95% CI (0.176, 0.335); When OS was at a high level (+1 SD), the conditional indirect effect was β = 0.321, 95% CI (0.234, 0.415). None of the three confidence intervals included zero, indicating that the indirect effect was significant at different OS levels. Further calculation of the Index of Moderated Mediation (IMM) showed that β = 0.074, 95% CI (0.022, 0.132), *p* = 0.006, indicating that OS significantly enhanced the indirect pathway of PO acting on TI through AI. In contrast, PI did not show a significant moderating effect on the “AI → TI” mediating pathway, with a moderating mediating index of β = 0.042, 95% CI (−0.006, 0.101), *p* = 0.088, not reaching a significant level.

**Table 5:** Moderated mediation analysis.

Indicator	OS level	Conditional indirect effect β	Lower limit of 95% CI	Upper 95% CI	Significance
Conditional indirect effects (PO → AI → TI)	Low (−1 SD)	0.174	0.102	0.256	Significant
	Mean	0.248	0.176	0.335	Significant
	High (+1 SD)	0.321	0.234	0.415	Significant
Moderating mediating index (IMM)	OS regulation	0.074	0.022	0.132	Significant
Moderated mediating index (IMM)	PI regulation	0.042	−0.006	0.101	Not significant

As shown in Table 6, the overall effect decomposition results indicate that the direct effect of PO on TI is not significant (β = 0.094, *p* = 0.204), but its indirect effect through AI is significant [β = 0.248, *p* < 0.001, 95% CI (0.176, 0.335)]. Therefore, the overall effect of PO on TI was significantly positive [β = 0.342, 95% CI (0.251, 0.433)]. The results show that Psychological Ownership of Research mainly relies on the mediating role of Academic Identity to promote Teaching Innovation, presenting a completely mediating feature.

**Table 6:** Decomposition of total effects.

Paths	Direct effect β (*p*)	Indirect effects β (95% CI)	Total effect β (95% CI)	Conclusion
PO → TI	0.094 (*p =* 0.204)	0.248^***^ (0.176, 0.335)	0.342^***^ (0.251, 0.433)	Indirect predisposition, complete mediating tendency

^***^p < 0.001

### 4.3 Multi-group analysis

To test the differences in model paths among different types of university teachers, this study divided the sample into three groups: research universities, applied universities, and vocational colleges, and used Multi-Group Analysis (MGA) to compare the critical paths.

As shown in Table 7, the PO → AI path was strongest in the research university sample (β = 0.526, *p* < 0.001), at a medium level in the applied university sample (β = 0.463, *p* < 0.001), and slightly lower in the vocational college sample (β = 0.398, *p* < 0.01). The cross-group difference test shows that there is a significant difference in the path coefficient between research-oriented universities and vocational colleges (Δβ = 0.128, *p* = 0.031), indicating that Psychological Ownership of Research is more likely to be transformed into Academic Identity among teachers in research-oriented universities.

**Table 7:** Results of multi-group analysis.

Paths	Research universities β	Applied university β	Higher vocational colleges β	Significant differences
PO → AI	0.526^***^	0.463^***^	0.398^**^	Δβ (research-oriented—vocational) = 0.128^*^, *p =* 0.031
AI → TI	0.521^***^	0.508^**^	0.492^**^	Not significant
OS × PO → AI	0.161^**^	0.128^*^	0.097	Δβ (research-oriented—vocational) = 0.064, *p =* 0.092

^*^p < 0.05, ^**^p < 0.01, ^***^p < 0.001.

On the path from AI to TI, all three types of universities showed significant positive effects, but the coefficient differences were not significant (research-oriented β = 0.521; application-oriented β = 0.508; higher vocational β = 0.492, p difference > 0.10), indicating that the promoting effect of Academic Identity on Teaching Innovation is universal.

On the OS × PO → AI moderating path, the moderating effect was most pronounced in the sample of research universities (β = 0.161, *p* < 0.01), while higher vocational colleges were relatively weaker (β = 0.097, *p* = 0.082). The margin of cross-group difference was significant (Δβ = 0.064, *p* = 0.092). This suggests that the role of Perceived Organizational Support varies among different types of institutions, especially in research-oriented universities, where it can amplify the positive effect of Psychological Ownership of Research.

## 5 Conclusions

### 5.1 Discussion

This study examined how Psychological Ownership of Research influences Teaching Innovation through Academic Identity and further explored the boundary roles of Perceived Organizational Support and Professional Identity. The results indicate that Psychological Ownership of Research did not directly translate into Teaching Innovation, but operated through Academic Identity. Rather than being a merely statistical pattern, this finding suggests that research related psychological investment does not automatically become innovative teaching practice. Although prior research has often assumed that research engagement enriches teaching through updated knowledge, methodological transfer, or stronger instructional confidence (Hu and Kuh, 2002), the present findings indicate that such a relationship may remain incomplete unless teachers first incorporate research into their academic self understanding.

More importantly, the full mediation result helps clarify why identity is indispensable in the research teaching nexus. Psychological ownership may generate responsibility, control, and personal attachment to research, but these feelings can remain confined to the research domain unless they are transformed into a role based understanding of the self. In this sense, Academic Identity functions as the mechanism through which research related ownership becomes pedagogically meaningful. Teachers do not innovate in teaching simply because they care about research; they are more likely to do so when they come to understand themselves as academic actors who integrate the roles of researcher and educator. This interpretation extends existing identity based explanations by showing that the key issue is not merely whether teachers engage in research, but whether research becomes part of how they define their academic role in relation to teaching (Billot, 2010; Henkel, 2005; Marschall, 2022; Cloonan et al., 2019).

Regarding boundary conditions, the results show that Perceived Organizational Support significantly strengthened the relationship between Psychological Ownership of Research and Academic Identity, whereas Professional Identity did not significantly moderate the relationship between Academic Identity and Teaching Innovation. The significant role of organizational support is consistent with the view that identity formation is not purely an internal process but is shaped by whether institutions provide recognition, resources, and legitimacy for combining research with teaching (Shen et al., 2024; Hassan et al., 2024). When organizational support is salient, teachers are more likely to interpret research ownership as institutionally valued and therefore more readily integrate it into their academic identity.

By contrast, the non significant moderating effect of Professional Identity should not be treated as incidental. A theoretically meaningful interpretation is that Professional Identity and Academic Identity operate at different levels. Professional Identity reflects a broader commitment to teaching as a valued profession, whereas Academic Identity more directly captures the integration of researcher and educator roles. As a result, general commitment to the teaching profession may not be sufficient to strengthen the conversion of Academic Identity into specific innovative teaching behaviors. Another possible explanation is that Professional Identity in this sample may function more as a normative or value based orientation than as an action activating condition. Teachers may strongly value the profession of teaching, yet such value commitment does not necessarily determine whether they redesign courses, integrate research into instruction, or experiment with new pedagogical tools. In addition, given the conceptual proximity between Academic Identity and Professional Identity, some degree of overlap in self reported perceptions may also have reduced the observable moderating effect, even though the statistical tests supported discriminant validity. Taken together, the non significant finding suggests that not all identity related constructs play the same role in the research teaching nexus, and that role integration may matter more than general professional commitment when explaining how research based psychological resources are translated into teaching innovation.

The multi group analysis further suggests that the transformation from Psychological Ownership of Research to Academic Identity is more pronounced in research universities than in vocational colleges. This pattern is theoretically informative because it implies that the meaning of research ownership is partly shaped by institutional context. In research intensive environments, research activity is more strongly embedded in evaluation systems, peer recognition, and everyday academic interaction. Under such conditions, teachers who feel strong ownership of research are more likely to view that ownership as central to who they are as academics, thereby strengthening the path from Psychological Ownership of Research to Academic Identity. By contrast, in vocational colleges, where teaching practice and applied training may be more institutionally emphasized, research ownership may be less readily legitimized as a defining basis for academic self construction.

A similar logic may explain why the moderating role of Perceived Organizational Support was more visible in research universities. In such settings, organizational support does not merely provide resources; it also signals that integrating research and teaching is institutionally valued. This makes it easier for teachers to convert research ownership into a stable academic identity. Therefore, the multi group findings suggest that the research teaching nexus is not driven by individual psychology alone. Rather, institutional role expectations and organizational signals shape whether research based psychological attachment can develop into identity and, ultimately, into innovative teaching practice.

Taken together, these findings suggest that the research teaching nexus should be understood less as a direct spillover from research activity to classroom practice and more as an identity based transformation process shaped by organizational context.

Overall, the conclusions of this study have three critical implications. Firstly, it breaks through the limitations of the “knowledge transfer” perspective and emphasizes that identity construction is the core mechanism for scientific research-driven Teaching Innovation. Secondly, it reminds us that organizational support is more context-sensitive than Professional Identity and can directly affect the transformation process between teachers‘ psychology and identity. Thirdly, it reveals the differences in the intensity of transformation between Psychological Ownership of Research and Academic Identity among different types of institutions, suggesting that future research should pay more attention to the interaction between institutions and culture. Overall, this study not only deepens the theoretical understanding of the integration mechanism of science and education, but also provides new ideas for enhancing teachers' Teaching Innovation and educational quality in practice.

### 5.2 Research contributions

First, this study breaks through the long-standing simple linear assumption about “research promoting teaching”. Previous studies mostly regarded the role of scientific research in teaching as a direct transfer of knowledge and methods (Brew, 2010; Tight, 2016). However, this study found that the influence of Psychological Ownership of Research on Teaching Innovation was mainly achieved indirectly through Academic Identity, and the direct effect was not significant. This discovery responds to the concern about the “identity transformation” mechanism in recent higher education research (Kaasila et al., 2021), indicating that for teachers to achieve the true integration of scientific research and teaching, they need to go through the process of Academic Identity reconstruction. The results not only enrich the application of psychological ownership theory in the educational field, but also provide new empirical support for identity theory in explaining the relationship between research and teaching.

Secondly, this study constructs and validates a comprehensive model that includes mediating effects, moderating effects, and moderating mediating effects, and compares the differences among different types of institutions through multiple sets of analyses. This multi-level model setting, which is relatively rare in higher education research, helps to reveal the complex mechanism of interaction between individual psychology—identity construction—teaching behavior. By incorporating Perceived Organizational Support and Professional Identity, this study not only verified the significance of external situational variables but also revealed their differentiated mechanisms of action, providing an example for the introduction of situational interaction perspectives in educational research in the future.

Finally, the findings of this study have practical implications for the development of college teachers and the improvement of educational quality. The conclusion indicates that relying solely on the individual scientific research psychological motivation of teachers is insufficient to promote Teaching Innovation. It is necessary to attach importance to the construction of Academic Identity and the role of institutional support. Colleges and universities should provide teachers with a supportive environment that combines scientific research and teaching through policy guidance and resource allocation, thereby enhancing the identity transformation efficiency of Psychological Ownership of Research. This has direct value for promoting Teaching Innovation among teachers in different types of institutions and improving the overall quality of education.

### 5.3 Future research directions

Although this study has achieved certain theoretical and empirical results, there are still several limitations. Future research can be further expanded in the following aspects.

First, this study relied on self-report questionnaires, which may involve social desirability bias and may inflate some observed relationships. Although Harman's single-factor test and the ULMC approach suggest that common method variance does not appear to be a major concern, it cannot be fully ruled out. Future research could combine self-report measures with additional sources such as student evaluations, peer observations, classroom records, or other objective indicators of teaching practice to strengthen the robustness of the findings.

Second, this study focuses on Psychological Ownership of Research, Academic Identity, Perceived Organizational Support and Professional Identity, but other potential variables may also affect teachers' Teaching Innovation. For example, factors such as academic stress, research evaluation system, teaching autonomy, and interdisciplinary collaboration may all play significant roles in different contexts (Shin and Jung, 2014). Future research could incorporate these variables into models to further reveal the multiple pathways of psychological and behavioral transformation of teachers.

Third, although the sample covers multiple types of higher education institutions in China, the findings should still be interpreted within a specific cultural and institutional context. The structure of the research teaching relationship, the meaning of academic identity, and the role of organizational support may vary across national systems of higher education. Therefore, the external validity of the present findings may be limited, and broad generalizations to global higher education should be made with caution. Future studies could test the applicability of the model in cross national or cross regional samples and compare whether the same mechanisms hold under different institutional logics, especially across emerging and developed higher education systems.

Finally, the cross-sectional data used in this study makes it difficult to capture the dynamic evolution between teachers' psychological ownership, academic identity and teaching innovation. Future studies could use longitudinal tracking designs or qualitative research methods to reveal how scientific psychological ownership changes with teacher career development stages and how it influences teaching innovation through identity construction at different career stages.

## Data Availability

The raw data supporting the conclusions of this article will be made available by the authors, without undue reservation.
